# Genomic Insights into the Population Structure and Antimicrobial Resistance of the Most Prevalent Human-Associated Non-Typhoidal *Salmonella* Serotypes in Romania

**DOI:** 10.3390/microorganisms14081716

**Published:** 2026-08-05

**Authors:** Mihaela Oprea, Laura-Ioana Popa, Daniela Cristea, Sorin Dinu, Andreea Ghiță, Catherine Ragimbeau, Lavinia-Cipriana Rusu, Codruța-Romanița Usein

**Affiliations:** 1 Cantacuzino National Military Medical Institute for Research and Development, 103 Splaiul Independentei, 050096 Bucharest, Romania; oprea.mihaela@cantacuzino.ro (M.O.); popa.laura@cantacuzino.ro (L.-I.P.); cristea.daniela@cantacuzino.ro (D.C.); iolea.andreea@cantacuzino.ro (A.G.); codrutausein@gmail.com (C.-R.U.); 2 Luxembourg National Health Laboratory, 1 Rue Louis Rech, L-3555 Dudelange, Luxembourg; catherine.ragimbeau@lns.etat.lu; 3 National Institute of Public Health, 1–3 Dr. Leonte Anastasievici Street, 050463 Bucharest, Romania; lavinia.rusu@insp.gov.ro

**Keywords:** *Salmonella enterica*, Enteritidis, Typhimurium, monophasic Typhimurium, WGS, AMR, Romania

## Abstract

This study aimed to identify circulating lineages of the most prevalent human-associated *Salmonella* serotypes identified through routine surveillance in Romania between 2023 and 2025 and assess their role in the spread of antimicrobial resistance (AMR). A total of 271 isolates (217 *S.* Enteritidis, 28 monophasic *S.* Typhimurium, and 26 *S.* Typhimurium) were investigated using Illumina short-read sequencing. The isolates were assigned to 9 MLST sequence types (STs) and 109 core genome MLST complex types. The predominant STs were ST11 (96% in *S.* Enteritidis), ST19 (77% in *S.* Typhimurium), and ST34 (79% in monophasic *S.* Typhimurium). Overall, 148 isolates carried at least one AMR determinant, including 28 acquired genes and 6 gene mutations, conferring resistance to ten antimicrobial classes. Four percent of all isolates were predicted to be multidrug-resistant, often associated with determinants conferring tolerances to biocides and/or heavy metals. The most prevalent resistance markers were GyrA_D87Y in *S.* Enteritidis ST11 (94/217) and *bla*_TEM-1_ in *S.* Typhimurium ST19 (7/26). Among monophasic *S.* Typhimurium ST34 isolates, the co-occurrence of *bla_TEM-1_*, *aph(3”)-Ib*, *aph(6)-Id*, *sul2*, and *tet(B)* genes was common (17/28). The 43 predicted AMR plasmids were not shared across serotypes, except among those carrying the *qnrB19* gene. These results provide a genomic baseline for AMR surveillance in Romania and strengthen the human surveillance component of national One Health efforts to control resistant *Salmonella*.

## 1. Introduction

Non-typhoidal *Salmonella* remains one of the leading causes of foodborne bacterial infections worldwide and represents a major public health concern due to the increasing emergence and dissemination of antimicrobial-resistant clones [1]. Effective surveillance of circulating clones is therefore essential for monitoring trends in antimicrobial resistance and supporting timely public health interventions. However, the capacity of laboratory-based surveillance systems varies considerably among countries, influencing their ability to detect, characterize, and monitor circulating *Salmonella* clones [2].

Whole-genome sequencing (WGS) is considered a state-of-the-art tool for infectious disease and drug resistance surveillance, providing high-resolution data needed for the novel precision public health approach [3].

Recognizing the importance of genomic surveillance, a strategic framework for strengthening national genomic surveillance capacity was established by the European Centre for Disease Prevention and Control (ECDC) [2], and WGS was credited by Regulation (EU) 2025/179 for epidemiological investigations of foodborne outbreaks at the EU level [4].

In Romania, WGS applications are underutilized, and no routine WGS-based surveillance program for bacterial pathogens is currently funded. As a result, the country is underrepresented in EU-wide efforts to implement effective genomic surveillance. However, initiatives have been undertaken to strengthen the research capacity of laboratories tasked with providing reference services for pathogens of public health importance. One key priority has been to improve national surveillance of zoonotic pathogens, which are considered major threats to public health. Since 2021, the laboratory responsible for reference functions has participated in several rounds of EU-organized WGS proficiency tests, providing a foundation for consistent generation of high-quality genomic data for foodborne pathogens from human hosts [5].

Building on accumulated experience and using widely adopted bioinformatics tools and databases, this study aimed to expand knowledge of isolates belonging to the three *Salmonella* serovars, most commonly recovered from humans in Romania: *Salmonella* Enteritidis, *S.* Typhimurium, and monophasic *S.* Typhimurium. The analysis was based on short-read sequencing and focused on contemporary isolates circulating in the country between January 2023 and August 2025, by combining bacterial subtyping with screening for antimicrobial resistance determinants and plasmids associated with their acquisition.

## 2. Materials and Methods

### 2.1. Salmonella Strains

A total of 271 strains were subjected to whole-genome sequencing. These strains, isolated from human clinical cases of salmonellosis diagnosed between 2023 and 2025 across 25 counties of Romania, were submitted to the Cantacuzino National Military Medical Institute for Research and Development (Cantacuzino Institute) by public health laboratories and Romanian hospitals during laboratory surveillance activities. The institute hosts a Reference Center (RC) that provides reference laboratory services for *Salmonella* and other enteric bacterial pathogens for both the public and private sectors and actively participates in ECDC-organized surveillance programs for gastrointestinal infections. The study strains were initially serotyped, using conventional agglutination and classified as *S.* Enteritidis (217 strains), *S.* Typhimurium (26 strains), and its monophasic variant 1,4,[5],12:i:- (28 strains). Epidemiological information on the clinical isolates tested, drawn from laboratory records, was limited to the geographical location of the clinical cases at the county level, the month/year of collection, demographic characteristics (age and sex), and, when available, limited information on outbreak association. Details of all the strains used in this study are provided in Table S1.

### 2.2. Phenotypic Antimicrobial Susceptibility Testing

Antimicrobial susceptibility testing was performed using the disk diffusion method in accordance with the European Committee on Antimicrobial Susceptibility Testing (EUCAST) guidelines and Clinical Breakpoint Tables, version 16.0 [6]. The antimicrobial panel included ampicillin, amoxicillin–clavulanic acid, gentamicin, cefotaxime, ceftazidime, pefloxacin, trimethoprim–sulfamethoxazole, and meropenem.

### 2.3. DNA Extraction

DNA was extracted automatically from fresh overnight cultures of *Salmonella* on a Maxwell CSC instrument using the Maxwell RSC Cultured Cells DNA Kit (Promega, Madison, WI, USA) or manually with the PureLink™ Genomic DNA Mini Kit (Invitrogen, Carlsbad, CA, USA), following the manufacturers’ instructions.

### 2.4. Whole-Genome Sequencing

Libraries were prepared using the Nextera XT DNA Library Preparation Kit (Illumina, San Diego, CA, USA), and sequencing was performed on Illumina MiSeq or NovaSeq 6000 platforms, using 2 × 250 bp or 2 × 150 bp paired-end chemistry, respectively. The quality of the sequences was assessed according to the criteria recommended by the FWD AMR-RefLabCap, an EU project designed to support ECDC activities and ensure the effective operation of microbiology reference laboratories for AMR testing and surveillance in *Salmonella* and *Campylobacter* [7].

### 2.5. Bioinformatics Analysis

Bruker MBioSeq Ridom Typer software vers. 11.0. (Ridom GmbH, Münster, Germany), formerly known as Ridom SeqSphere+, with the included tools was used for the automatic processing and analysis of Illumina raw reads, which were assembled using the SKESA 2.4.0 algorithm. Specifically, in silico serotyping analysis was performed with the *Salmonella* in silico Typing Resource (SISTR) tool, the detection of antimicrobial resistance-associated genes and point mutations with the NCBI AMRFinderPlus tool (thresholds: 90% identity and 99% reference coverage), and the reconstruction and typing of plasmid sequences with the MOB-suite tool (identity 90%, alignment to reference 99% thresholds). The software was also used to determine *Salmonella*-specific sequence types (STs) according to Achtman’s 7-locus multilocus sequence typing (MLST) scheme [8], and complex types (CTs) were defined based on the 3002 loci included in the EnteroBase core genome MLST (cgMLST) v2 scheme [9]. Of note, due to a slightly different allele calling procedure, the allele numbering provided by the software was independent of and incompatible with the EnteroBase allele nomenclature.

Minimum spanning trees were constructed based on comparisons of allele variants across isolates to facilitate the investigation of closely related isolates. For all serotypes, cluster identification, denoting high genetic relatedness among at least two isolates and clinical cases likely to be epidemiologically linked, was performed at ≤5 allelic differences, a threshold used in outbreak investigations in Europe [10]. To refine cluster identification for *S.* Enteritidis, the cluster alert threshold was lowered to ≤3 allele differences.

## 3. Results

### 3.1. Sequencing Parameters and Data Availability

All 271 strains met the quality control metrics and produced sufficient data to generate confident draft genomes assemblies for further analysis. Estimated draft genome sizes ranged from 4.6 to 5.2 Mbp, with mean sequencing depths ranging from 47× to 186×, a maximum of 375 contigs, and N50 values exceeding 33,851 bp (mean 79,065 bp) (Table S1). The sequencing data were deposited in the European Nucleotide Archive (ENA) under Projects PRJEB40400 and PRJEB89723.

### 3.2. In Silico Serotype and ST Identification

The WGS-predicted serotypes confirmed the accuracy of the antigenic formula previously assigned to each strain by traditional agglutination. Overall, in silico MLST assigned the Romanian *Salmonella* strains to nine STs, of which only three (i.e., ST11, ST19, and ST34) persisted throughout the study period.

The cgMLST analysis further provided higher-resolution typing, assigning them to 109 CTs (Table S2).

Most *S.* Enteritidis strains were classified as ST11 (208/217 strains, 96%), followed by its single-locus variants (SLVs) ST11737 (6 strains) and ST3233 (3 strains). The ST11 strains were split into 67 CTs. *S.* Typhimurium strains were distributed across ST19 (20 strains) and its SLVs ST10377 (2 strains), ST568 (3 strains), and ST12075 (1 strain), while the monophasic *S.* Typhimurium strains were assigned to the ST34 (22 strains) and ST19 (6 strains) lineages. For the biphasic and monophasic *S.* Typhimurium ST19 strains, cgMLST provided 13 and 5 distinct profiles, respectively. The 22 monophasic *S.* Typhimurium strains derived from ST34 were classified into 17 CTs.

### 3.3. Identification of Antimicrobial and Biocide Resistance Determinants and Their Distribution Across the Romanian Salmonella Strains

Of the 271 strains examined, 148 (54.6%) were predicted by AMRFinderPlus to carry at least one acquired antimicrobial resistance gene and/or gene modification conferring resistance to 10 antimicrobial drug classes, and 4% of these strains were genotypically predicted to be resistant to at least 3 antimicrobial drug classes, thereby qualifying for MDR potential. Overall, strains harboring determinants associated with resistance to quinolones (43.1%), beta-lactams (10.3%), aminoglycosides (8.8%), sulfonamides (8.8%), tetracyclines (8.4%), phenicols (1.8%), trimethoprim (1.8%), macrolides (1.4%), colistin (0.3%), and bleomycin (0.3%) were identified. The most frequent were the *gyrA* mutants (40.9%), followed by *bla_TEM-1_* (9.9%), *aph(6)-Id* (8.1%), *aph(3″)-Ib* (8.1%), *sul2* (8.4%), and *tet(B)* (6.6%).

Overall, the presence of antimicrobial resistance determinants showed good concordance with the phenotypic antimicrobial susceptibility results, with only a few exceptions: one *cmlA1-*positive strain exhibited a susceptible phenotype to phenicols, but upon a more detailed analysis of the gene, an internal deletion was observed, likely affecting the gene’s function; phenotypic resistance to ampicillin and amoxicillin–clavulanic acid could not be explained for one strain, and one GyrA_D87Y-positive strain retained the phenotype susceptible to quinolones. However, for several antimicrobials (i.e., tetracycline, macrolides, and streptomycin), genotype–phenotype correlations could not be assessed because the testing panel did not include the corresponding antimicrobial agents. Additionally, two genes conferring tolerance to biocides (i.e., *qacEdelta1* and *qacL*) and six metal operons (i.e., arsenic, copper, gold, mercury, silver, and tellurite) were observed across the genomes screened. While one or both biocide resistance genes were found in only 1.8% of strains, individual genes of metal operons ranged in prevalence from 100% (i.e., *golS*, *golT*) to 1.1% (i.e., *terD*, *terW*, *terZ*).

An overview of the AMR markers identified in Romanian strains grouped by antimicrobial type and classes is provided in Table 1.

Differences were observed among the three serotypes in terms of genetically predicted AMR load and spectrum of AMR drug resistance.

Of the 217 *S.* Enteritidis strains, 115 (52.9%) carried single genes or gene mutations conferring AMR. Single-point mutations in the quinolone resistance-determining region (QRDR) of the *gyrA* gene, with most at codon 87, predominantly changing aspartic acid to tyrosine (GyrA_D87Y), were the most common (110 strains). Less frequently detected were the plasmid-mediated quinolone resistance gene *qnrB19* (in two strains), the mutated GyrB_E466D and AcrB_R717L genes (in one isolate each), and the *bla*_TEM-1_ gene (in one strain).

In the *S.* Typhimurium subset, 10 of the 26 strains (38.4%) were predicted to harbor AMR genes. Six strains carried only *bla*_TEM-1_, and the remaining four had MDR genotypes with inferred partially overlapping resistance while being supported by different gene combinations, namely streptomycin (*aph(3″)-Ib*, *aph(6)-Id*), sulfonamide (*sul*2), and tetracycline (*tetA*) or ampicillin (*bla*_TEM-1_), streptomycin (*aadA*1), chloramphenicol (*cmlA*1), and tetracycline (*tetA*).

The monophasic variant of *S.* Typhimurium had elevated AMR gene prevalence, with 24 of 28 strains (85.7%) predicted to be resistant and have between 1 and 14 genes per genome. With 26 unique genes spanning resistance to 10 antibiotic classes, including aminoglycosides, beta-lactams, macrolides, phenicols, sulfonamides, trimethoprim, tetracyclines, quinolones, colistin, and glycopeptides, the monophasic *S.* Typhimurium strains were characterized by 12 AMR genotypes. Nine of them were suggestive of MDR phenotypes, and the most commonly detected ones comprised exclusively the association of the *bla_TEM-_*_1_/*aph(3″)-Ib*/*aph(6)-Id*/*sul2*/*tetB* genes (11 strains). This combination conferred a specific four-drug resistance to ampicillin, streptomycin, sulfonamides, and tetracyclines, commonly known by the abbreviation ASSuT. In fact, this ASSuT gene core was identified in 17 monophasic *S.* Typhimurium strains, of which 6 contained additional AMR genes. Aminoglycoside resistance genes as a group were the most diverse in monophasic *S.* Typhimurium, encoding aminoglycoside phosphotransferases (*aph(3″)-Ib*, *aph(6)-Id*, *aph(4)-Ia*, *aph(3′)-Ia*), nucleotidyltransferases (*aadA1*, *aadA2*, *aadA5*, *ant(2”)-Ia*), and acetyltransferases (*aac(3)-IVa*, *sat2*). Genes associated with resistance to antimicrobial drugs such as azithromycin (*mef(B)*, *mphA*), quinolones (*qnrB19*), trimethoprim (*dfrA1*, *dfrA1*2, *dfrA19*), and bleomycin (*bleO*), as well as the *mcr-9.1* gene controversially associated with colistin resistance, were also found, contributing to the genetic variability within serotypes and separation from biphasic *S.* Typhimurium in terms of resistance-conferring genes and mutations. The WGS-based AMR genotypes assigned to the Romanian *Salmonella* strains in this study, in correlation with their serotypes and STs, are summarized in Table 2 and detailed in Table S2.

Biocide and metal resistance genes were found predominantly in monophasic *S.* Typhimurium strains, except for the gold operon genes *golS* and *golT*, which were present in all strains regardless of serotype, as expected, being part of the conserved core genome of *S. enterica* subsp., with the exception of the human-adapted serotypes *S*. Typhi and *S*. Paratyphi A [11]. The co-occurrence of operons encoding resistance to arsenic, copper, silver, and mercury was predicted in most monophasic *S.* Typhimurium ST34 strains, whereas these operons were absent in *S.* Enteritidis and *S.* Typhimurium. Although rare, tellurite resistance genes were also predicted exclusively in ST34 isolates. Regarding biocide resistance genes, *qacL* and/or *qacEdelta1* were predicted in four monophasic and one biphasic *S.* Typhimurium strains, but not in *S.* Enteritidis.

### 3.4. Cluster Analysis of S. *Enteritidis*, S. *Typhimurium*, and Monophasic S. *Typhimurium* Strains

All 217 *S.* Enteritidis strains subjected to allele-based cluster analysis fulfilled “good target” quality control for ≥ 98% of the scheme’s 3002 target loci. Therefore, the cgMLST typing results were visualized in minimum spanning trees (MSTs).

An initial cgMLST-based cluster analysis using a five-allelic difference (AD) threshold for cluster definition revealed 17 clusters comprising 2 to 90 strains and 38 singletons. Due to chaining, within the 90-strain cluster, the allele range expanded from 0 to 40 alleles.

When the AD threshold was set to 3, the number of clusters increased to 31, and their size range changed from 2 to 19 strains. These clusters comprised 166 (76.5%) of the 217 *S.* Enteritidis strains, with the remaining strains left outside a cluster. Twenty-nine distinct subclusters were defined, all with pairwise distances of 0 alleles. The ADs separating singletons from the nearest neighboring strain ranged from 4 to 147 alleles. In the new MST, the largest 90-strain cluster was split into 13 clusters (Figure S1).

When the data on GyrA mutations were overlaid on the MST (3 AD), strains that had such mutations did not intermingle with those without mutations. Specifically, 80 of the 94 strains with the single mutation GyrA_D87Y formed 17 clusters and 11 subclusters, and 14 strains were singletons. The cgMLST data also clustered 12 of the 15 strains with the GyrA_S83Y mutation, leaving only three outliers. The remaining strains, most of which harbored no AMR determinants, were grouped into 13 clusters with sizes varying from 2 to 14 strains, and 36 strains were left outside these clusters. Scattered among the latter were the following strains: a strain with the single GyrA_D87N substitution, a strain carrying only a GyrB_E466D mutation, a strain carrying the AcrB_R717L gene mutation, and two *qnrB19*-bearing strains.

Overall, the *S.* Enteritidis clusters included strains from the same outbreak as well as strains without a known epidemiological link, as detailed in Table S1.

The MST (5 AD) generated jointly for the biphasic and monophasic *S.* Typhimurium strains revealed that 19 strains were distributed across seven clusters with between two and five members, and 35 strains were singletons (Figure S2). The retrospective epidemiological data available indicated that only eight strains were known to be outbreak-associated (Table S1). Within each cluster, the strains belonged to the same ST, and biphasic *S.* Typhimurium did not cluster with the monophasic variants. Four clusters, designated MST Clusters 1, 3, 6, and 7, comprised 12 *S.* Typhimurium strains. Only four of these genetically closely related strains were associated with data supporting epidemiological links between cases of origin, which were included in C1 and C7 clusters. Three clusters, designated MST Clusters 2, 4, and 5, were formed by seven monophasic *S.* Typhimurium strains. Only the strains in cluster C5 were epidemiologically linked.

### 3.5. Predicted Plasmids in Romanian Salmonella Strains

The approach relied solely on MOB-suite tools to identify resistance gene-bearing plasmids from short-read WGS data. In total, 417 plasmid-like assemblies were found in 266 of the 271 strains investigated, with individual strains carrying between one and eight. AMRFinderPlus detected at least one gene conferring resistance in only 43 of the 417 assemblies (10.3%) treated as plasmids. These presumptive AMR plasmids were associated with 22 monophasic *S.* Typhimurium, 9 biphasic *S.* Typhimurium, and 2 *S.* Enteritidis genomes. Not exceeding 3 per genome, they had sizes estimated between 2699 bp and 273,697 bp. Considering three size categories, 13 plasmids were classified as “small” (<10 kbp), 23 as “medium” (10–50 kbp), and 7 as “large” (>50 kbp). Almost all small plasmids (12/13) were reconstructed from single contigs, whereas medium and large ones were from a median number of contigs of 3 and 13, respectively.

**Figure 1:**
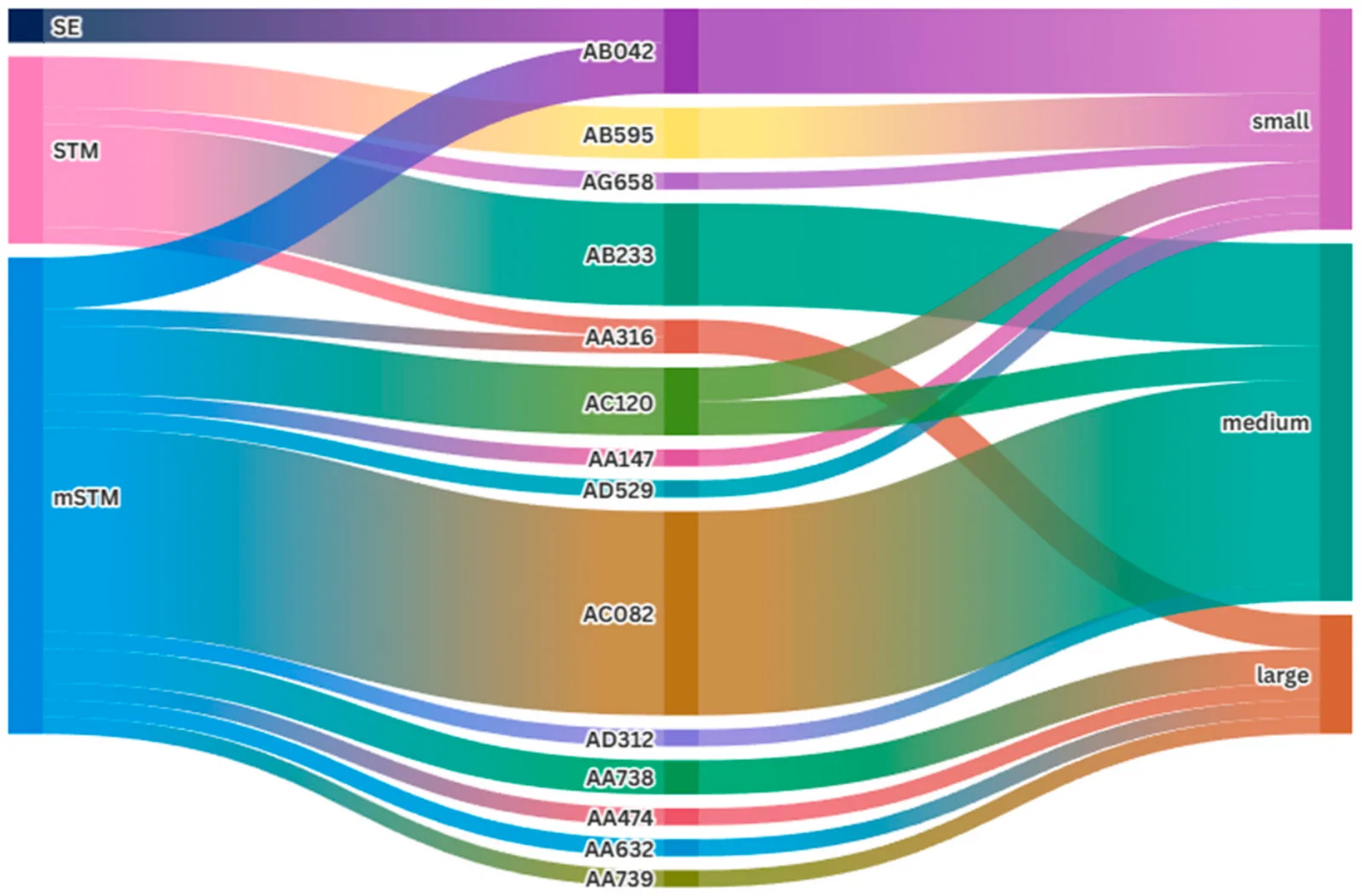
Distribution by serotype, according to MOB-cluster and size, of the plasmids predicted to confer resistance to the Romanian *Salmonella* strains. Plasmid reconstruction and typing with MOB-suite tool. Sankey diagram for data visualization on Flourish platform (https://flourish.studio, accessed on 7 May 2026). SE = *Salmonella* Enteritidis, STM = *Salmonella* Typhimurium, mSTM = monophasic *Salmonella* Typhimurium.

MOB-typer also provided a primary cluster ID based on a MASH distance cut-off of 0.06, separating the 43 AMR plasmids into 14 MOB-suite plasmid clusters, of which 8 were assigned one plasmid each, and the remaining were represented by at least 2 plasmids. The most common MOB-clusters were AB042 (n = 5), AB233 (n = 6), and AC082 (n = 12), accounting for 23 of the 43 plasmids (53.5%). AB042 MOB-cluster comprised plasmids from both *S.* Enteritidis and monophasic *S.* Typhimurium, carrying the *qnrB19* gene and having similar sizes of approximately 2.3 kb. The AA316 MOB-cluster was shared by both variants of the *S.* Typhimurium serotype on large conjugative plasmids, although they carried different cargo genes. The serotype distribution of the reconstructed plasmids in relation to their size and classification in MOB-clusters is presented in Figure 1.

**Figure 2:**
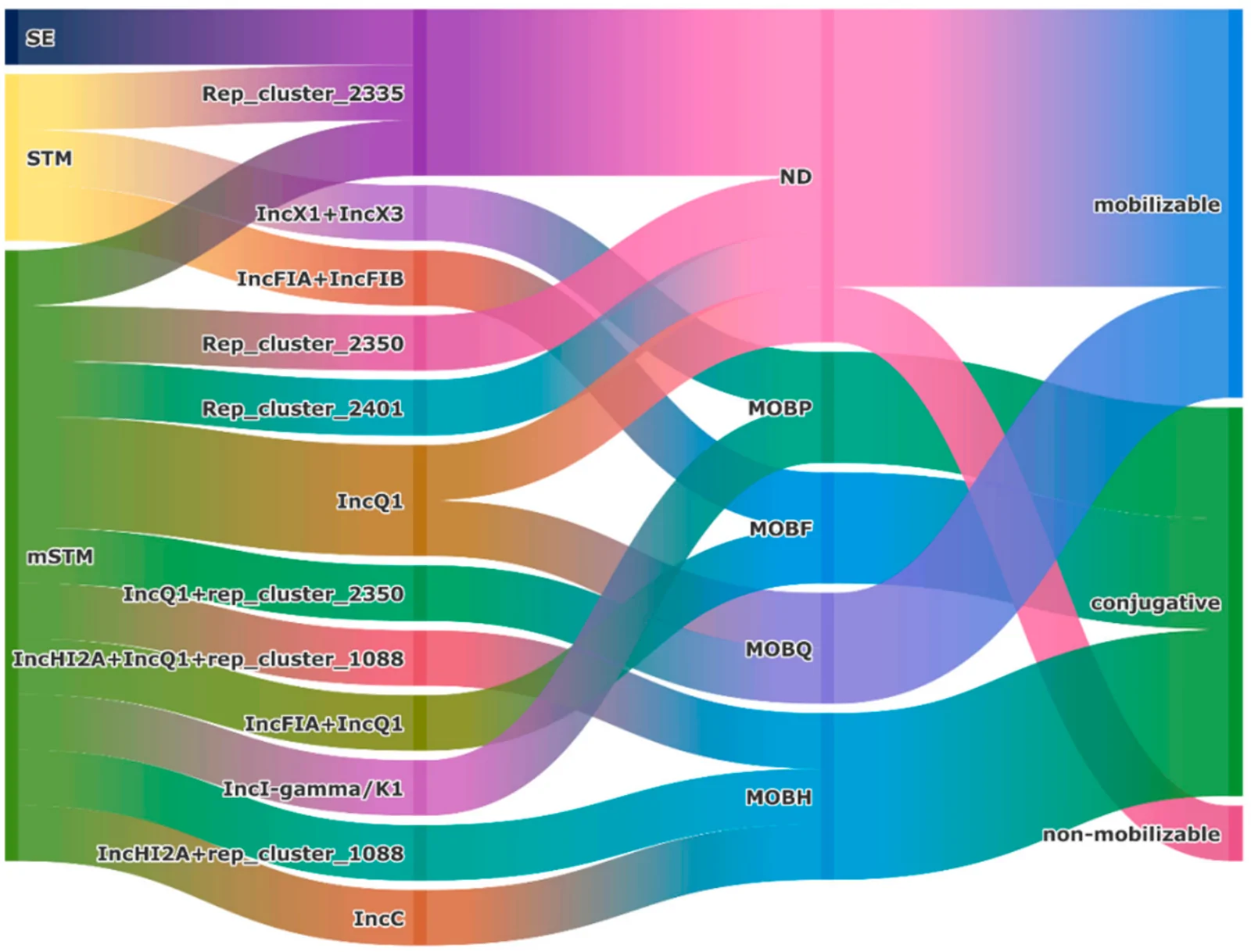
Distribution by serotype, according to replicon type(s), relaxase type, and mobility, of the plasmids predicted to confer resistance to the Romanian *Salmonella* strains; plasmid reconstruction and typing performed with the MOB-suite tool. Sankey diagram for data visualization on Flourish platform (https://flourish.studio, accessed on 7 May 2026). SE = *Salmonella* Enteritidis, STM = *Salmonella* Typhimurium, mSTM = monophasic *Salmonella* Typhimurium, ND = not determined.

MOB-typer provided information on the replicon type for 40 of the 43 AMR plasmids (93%) and on the relaxase type for 19 plasmids (44.2%), and it was not able to type two plasmids. The mobility prediction report indicated that 16 plasmids were non-mobilizable, 14 were mobilizable, and 13 were conjugative. A summary of these plasmids’ typing features in association with the serotypes screened is presented in Figure 2.

AMRFinderPlus results showed that most of the acquired AMR genes found in this study were plasmid-encoded. The only genes that were not found on the reconstructed plasmids were *aadA5*, *bleO*, and *mcr-9.1*. In total, 17 plasmids harbored a single AMR gene (39.5%). Among these, MOB-cluster AB233 plasmids (IncX1, IncX3, MOBP, conjugative), about 45 kbp in length, with only the *bla_TEM-1_* gene, were most commonly detected. Twenty-six plasmids carried multiple ARGs with or without genes coding for tolerance to biocides and/or metals. Less conserved, based on the Mash distance within the cluster, MOB-cluster AC082 plasmids (IncQ1, non-mobilizable), with medium sizes ranging from 19.8 to 33.8 kbp, represented the most frequently shared plasmid group. All plasmids contained combinations of two to five of the following AMR genes: *bla_TEM-1_*, *aph(3″)-Ib*, *aph(6)-Id*, *sul2*, and *tetB*, with or without the *mer* operon.

The highest number of AMR genes was found in two large plasmids (273.7 kbp and 269.5 kbp in size), which were typed as IncHI2A, rep_cluster_1088, MOBH, conjugative, and assigned to MOB-cluster AA738. They were reconstructed from the genomes of two monophasic *S.* Typhimurium ST34 isolates assigned toCT 19765, isolated from single cases with no known epidemiological relationship. These plasmids contained 11 AMR genes conferring resistance to seven antimicrobial classes (i.e., aminoglycosides, beta-lactams, macrolides, sulfonamides, phenicols, tetracyclines, and trimethoprim), as well as genes encoding biocide and metal tolerance.

The fully automated association of plasmid typing information with AMR gene composition is presented in Table S3.

## 4. Discussion

In 2024, the European Observatory on Health Systems and Policies reported that, according to the 2023–2030 National Health Strategy, the AMR control was placed among the official public health priorities in Romania [12]. The country update addressed the continued threat posed by microbial resistance in Romania while affirming that national efforts are underway to heighten vigilance and engagement. In this context, strengthening the national microbiology laboratory system was a key focus in ensuring that AMR data are accurate and accessible for decision-making at all levels. Consequently, this study aimed to pave the way for integrating WGS into standard practice to detect emerging *Salmonella* resistance trends nationwide, given the constant national and international need for sharing data on zoonotic pathogens that can cause foodborne disease outbreaks.

Among culture-confirmed *Salmonella* isolates from human infections retrieved from the Romanian surveillance system between 2023 and 2025, *S.* Enteritidis was the leading contributor to human illness, followed by *S.* Typhimurium (including the monophasic variant), a trend observed for many years across European countries [13]. About three-quarters (76%) of the strains were *S.* Enteritidis ST11, indicating that since 2016, when it was first documented to circulate locally, this long-lived lineage, with a recognized propensity for causing prolonged multi-country outbreaks, has continued to propagate and cause morbidity in Romania [14]. In terms of antimicrobial resistance, 55% of these *S.* Enteritidis ST11 strains possessed genetic markers for AMR, which, with a few exceptions, were altered *gyrA* genes at codon S83 or D87, mutations commonly found in human isolates with high-level resistance to nalidixic acid [15]. Notably, the prevalence of GyrA mutants with D87Y substitution was markedly high, suggesting clonal expansion, a process previously considered to explain the high rates of *S.* EnteritidisEnteritidis quinolone resistance observed in other regions [16,17]. The concordance between genotypic AMR prediction and phenotypic testing was high, with only three discrepancies observed, supporting the usefulness of genotypic testing in public health AMR surveillance. Using the recommended cut-off as optimal for monitoring *S.* Enteritidis human outbreak clusters [18], the cgMLST-based cluster analysis provided data supporting this assumption. Considering the official veterinary reports and previous studies, locally sourced animal products could have contributed to the persistent exchange of these closely related *S.* Enteritidis organisms across counties in the country during the period studied [13,19,20].

The emergence of plasmid-borne *qnr* genes in *Enterobacteriaceae* has been documented in several Romanian studies that detected these genes by PCR-based approaches in isolates from humans, food, and animals [21,22,23]. Furthermore, isolates from a Romanian pig farm were linked to the first IncN-borne *qnrS1* gene described in porcine *Escherichia coli* in Europe [24]. While less common than resistance-associated mutations in the DNA gyrase gene, the plasmid-borne determinants detected in our study in both *S.* Enteritidis and monophasic *S.* Typhimurium isolates provide further evidence supporting the need to track their spread in our country [25].

Furthermore, although additional characterization is required, our findings provide the first evidence of the circulation of small MOB-cluster AB042 (replicon-type cluster 2335) plasmids harboring the *qnrB19* allele, highlighting the ongoing dissemination of such *qnr*-positive isolates in Romania. Previously described as located on different types of plasmids [26,27,28], *qnrB19* genes were demonstrated to confer unusually high-level quinolone resistance in the absence of any DNA gyrase mutation [29], as observed in the Romanian isolates, and in synergy with DNA gyrase mutations as well [30]. Moreover, the presence of the chromosomal AcrB_R717L mutation suggests a stable, non-plasmid-mediated mechanism contributing to macrolide resistance in these isolates.

Also included in the study collection were biphasic and monophasic *S.* Typhimurium clinical strains whose *draft* genomes have outlined, to our knowledge for the first time, a perspective on the *S.* Typhimurium population structure linked to human infections in Romania, revealing as major components the widely reported, multi-country outbreak-associated ST19 and ST34 clones [31]. While most *S.* Typhimurium clinical isolates were typed as ST19, a few strains were attributed to MLST types ST568, ST12075, and ST10377. Strains with STs matching these three SLVs of ST19 were searched by querying the EnteroBase database, a major repository of published *Salmonella* genomic data (January 2026) [32]. Hence, a total of 546 *S.* Typhimurium ST568 strains isolated from humans, animals, and the environment in various continents were identified. These included samples collected between 2023 and 2025 from patients in France, the United Kingdom (UK), Portugal, and Cyprus. The other two STs were represented by four ST12075 genome sequences of human isolates collected in Cyprus in 2023 and the UK in 2024, as well as three ST10377 non-human strains from feed and food collected between 2022 and 2025 in the UK and France. The inclusion of Romanian human isolate genome assemblies in the ST10377 dataset in EnteroBase showed that combining national surveillance datasets enhances the global understanding of the epidemiology and public health significance of this lineage.

The 3002-locus cgMLST scheme, already known for providing phylogenetically consistent and epidemiologically matching information sufficient in distinguishing outbreaks from sporadic *Salmonella* isolate [17,33,34,35], was used to maximize the differentiation of strains matched by the 7-gene MLST. Between 2023 and 2025, county public health authorities reported that most laboratory-confirmed *S.* Typhimurium infections were sporadic, rarely raising awareness of local outbreaks with few cases showing geographic clustering. This study revealed genetically closely related isolates from cases that were geographically and temporally dispersed, suggesting that transmission events had escaped detection because the strain-typing methods used in public health laboratories lacked the necessary resolution to distinguish linked infections from sporadic cases. These findings support the need to strengthen genomic surveillance of foodborne infections in Romania, using WGS-based surveillance data integrated with epidemiological information.

While determining the genetic types of *S.* Typhimurium that infected autochthonous human hosts during the recent period, the WGS-based AMR prediction illuminated those that contributed most to drug resistance. According to genomic data, in 2023–2025, ST34 had a higher proportion of isolates predicted to be resistant than ST19, and the less common STs in the dataset were represented by isolates predicted to be susceptible. AMR genetic markers were identified in 86% of the ST34 isolates, which, in the great majority, exemplified diversified multidrug resistance of monophasic *S.* Typhimurium already described in many regions. While combined resistance to drugs such as penicillins, aminoglycosides, sulfonamides, and tetracyclines was commonly detected, the associated genes *bla_TEM-1_*, *aph(3”)-Ib*, *aph(6)-Id*, *sul2*, and *tetB* were shared by most of these strains. This genetic configuration, which confers the so-called R-ASSuT (pheno)type, was demonstrated to be chromosomally located [36]. This configuration, most frequently disseminated by monophasic *S.* Typhimurium ST34 isolates, had become a genetic resistance fingerprint for the European ST34 clone, whose ASSuT-positive members received particular attention after causing two major outbreaks in Luxembourg [37]. Many of the ST34 strains recovered from the Romanian population likely derived from the same European lineage.

It should be noted that the detection of some low-prevalence plasmid-borne resistance genes in monophasic *S.* Typhimurium genomes posed additional clinical concerns. In addition to the low-prevalence *qnrB19* genes, the *mef(B)* and *mph(A)* genes were also of concern because they proved that plasmid-mediated resistance to macrolides such as azithromycin is arising in *Salmonella* serotypes circulating in Romania, and may compromise this antibiotic as a supporting option for severe cases of salmonellosis and eventually its usefulness for other public health pathogens [38].

It is worth noting that the *mph(A)* gene was identified in an ST34 isolate that displayed an AMR genotype comprising 12 drug resistance determinants, including the *mcr-9.1* variant of the mobile colistin resistance gene *mcr-9*. First reported in 2019 following in silico detection in a multidrug-resistant, colistin-susceptible *Salmonella enterica* serotype Typhimurium clinical isolate collected in 2010, the *mcr-9* gene was postulated to be one of the most abundant and disseminated members of the *mcr* family in human hosts [39,40]. In a recent descriptive study on the distribution of *mcr*-variants and *mcr*-carrying genomes deposited in the NCBI database, the *mcr*-9.1 gene ranked second in prevalence, being identified in 58% of the 3224 *mcr*-positive *S. enterica* available genomes [41]. Its geographical distribution showed that 33% of the genomes have been reported from European countries, among which Romania’s neighboring countries, Hungary and Serbia. The above-mentioned study provided an overview of the comprehensive NCBI reference genomic database, revealing a lower representation of positive genomes from Eastern Europe compared with other European regions. Western countries such as the United Kingdom, Germany, France, and the Netherlands were represented by the highest number of positive genomes. These findings most likely reflect differences in both the epidemiological context and the implementation of genomic surveillance across Europe, rather than the true distribution of the gene.

Our sequencing data are the first evidence of *mcr-9.1* in Romania, with the gene identified in a monophasic *S.* Typhimurium isolate from 2023. Therefore, given its known minimal clinical impact as a resistance determinant, we can speculate that such *mcr-9.1*-carrying *Salmonella* strains of human origin may circulate silently in the autochthonous population, and it will indeed be important to provide more microbiological information in the future to understand their epidemiological relevance, particularly given the scarcity of data from Eastern Europe [42].

The WGS data were used with the MOB-suite modular tools integrated into Bruker MBioSeq Ridom Typer to extract information on plasmids encoding antimicrobial resistance in clinically relevant human *Salmonella* isolates circulating in the country, which are largely understudied. The computational approach reconstructed and typed the plasmid sequences and compared them with reference plasmids from the MOB-suite database, which otherwise would not have been accomplished in our setting to cover the whole dataset. Furthermore, the option of MOB-typer cluster code assignment provided a plasmid classification framework suitable to be used as a basis for plasmid comparisons for epidemiological purposes [43].

The results presented here showed that the three serotypes investigated could be distinguished based on their plasmid profiles, except for the *qnrB19*-bearing mobilizable plasmids, which spread across serotypes. Data also highlighted that monophasic *S.* Typhimurium isolates with the co-occurrence of antibiotic resistance and heavy-metal tolerance determinants on the same plasmid are circulating in Romania [44]. Such plasmids were predicted to be conjugative and to belong to IncHI2A (in two different multi-replicon combinations) and IncC, with plasmid backbones appearing to facilitate the accumulation of a larger number of AMR genes in the monophasic *S.* Typhimurium serotype, highlighting their potential for inter-species dissemination and representing key information for risk assessment [45,46].

However, definitive statements cannot be made without further investigation because of the known challenges encountered when reconstructing AMR plasmids from short reads [47], as in our study. There is still some uncertainty about the identity of several plasmids, especially those reconstructed from many contigs. These should be further refined by combining short- and long-read sequences for potential correction. Reconstructing non-mobilizable plasmid sequences ranging from 14.2 to 33.8 kb that share the IncQ replicon gene and are associated with monophasic *S.* Typhimurium ST34 isolates presenting the ASSuT (pheno)type also requires a hybrid approach. The objective would be to identify the plasmid-derived genetic material cassette integrated into the chromosome, a typical feature among European ST34 isolates [36]. The pipeline currently used and presented in our study cannot unambiguously determine whether this specific cassette is located on a plasmid or integrated into the chromosome, particularly when assemblies are fragmented. Consequently, the plasmid reconstructions generated by MOB-suite should be interpreted as predictions rather than definitive evidence of gene localization. To overcome these limitations, we intend to implement a hybrid sequencing strategy combining Illumina short-read and Oxford Nanopore long-read sequencing in our future genomic surveillance activities. This approach will enable complete reconstruction of large plasmids, revealing the structural organization of AMR plasmids and the genomic context of resistance genes. Although a much clearer picture of the *S. enterica* pathogen emerged when WGS was performed on the Romanian clinical isolates, we acknowledge the limitations of our work. Challenged by the inherent functioning of the laboratory-based surveillance system, the convenience sampling approach impacted the volume of isolates studied, potentially hindering the genetic diversity, especially for the overall *S.* Typhimurium set. The unequal representation of the three serotypes in our study reflects the distribution of isolates received through the national surveillance system during the study period rather than a predefined sampling strategy, while also being consistent with both national and international findings [13]. This imbalance may limit direct comparisons between serotype characteristics; therefore such comparisons should be interpreted with caution.

Furthermore, the lack of complete metadata context made it difficult to analyze the links between isolates and plasmids, and precluded the full exploitation of the epidemiological value of the WGS data. Future national surveillance would greatly benefit from a standardized framework and methodology for integrating epidemiological metadata from clinical and public health laboratories with genomic data at the Reference Center. Such integration would facilitate the investigation of genetically related isolates without apparent epidemiological links, improve the detection of otherwise unrecognized transmission events, and support timely interventions to limit outbreaks.

## 5. Conclusions

Overall, the WGS data presented here and reflecting AMR characteristics for the three predominant *Salmonella* serotypes circulating in Romania in recent years, represent an important step toward the self-reliant genomic AMR surveillance in our region. Moreover, although our study included only human isolates, these genomic data provide an essential baseline that may facilitate future integration with animal, food, and environmental surveillance data within the One Health strategy promoted at EU level [48].

## Figures and Tables

**Table 1:** Distribution of the resistance determinants among the 271 *S. Enterica* isolates.

Type	Class	Gene/ Mutation	No. of Strains	Type	Class	Gene/ Mutation	No. of Strains
Drug	Aminoglycosides	*aph(3”)-Ib*	22	Biocide	Quaternary ammonium	*qacL*	4
		*aph(6)-Id*	22			*qacEdelta1*	2
		*aadA1*	5	Metal	Arsenic	*arsC*	21
		*aadA2*	3			*arsR*	21
		*aac(3)-IVa*	3			*arsA*	21
		*ant(2”)-Ia*	2			*arsB*	21
		*aph(4)-Ia*	3			*arsD*	21
		*aph(3′)-Ia*	3		Copper	*pcoR*	22
		*aadA5*	1			*pcoC*	22
		*sat2*	1			*pcoD*	22
	Beta-lactams	*bla_TEM-1_*	27			*pcoE*	23
		*bla_TEM-190_*	1			*pcoS*	22
	Macrolides	*mefB*	2		Gold	*golS*	271
		*mphA*	1			*golT*	271
		AcrB R717L	1		(Organo) Mercury	*merC*	20
	Phenicols	*cmlA1*	4			*merP*	17
		*floR*	1			*merR*	18
	Sulfonamides	*sul2*	23			*merT*	19
		*sul3*	4		Silver	*silA*	20
		*sul1*	2			*silB*	21
	Trimethoprim	*dfrA12*	3			*silC*	21
		*dfrA1*	1			*silE*	21
		*dfrA19*	1			*silF*	21
	Tetracyclines	*tet(A)*	3			*silR*	20
		*tet(B)*	19			*silS*	21
		*tet(M)*	1		Tellurium	*terD*	3
	Quinolones	GyrA D87Y	94			*terW*	3
		GyrA S83Y	15			*terZ*	3
		GyrA S83F	1				
		GyrA D87N	1				
		GyrB E466D	1				
		*qnrB19*	5				
	Polymixins (Colistin)	*mcr-9.1*	1				
	Glycopeptides (Bleomycin)	*bleO*	1				

**Table 2:** Distribution of the AMR profiles according to serotypes and STs in the Romanian *Salmonella* strain collection.

AMR Genotype	Serotype	ST	No. of Strains
GyrA_D87Y	SE	ST11	94
GyrA_S83Y	SE	ST11	15
GyrA_D87N	SE	ST11	1
GyrB_E466D	SE	ST11	1
AcrB_R717L	SE	ST11	1
*qnrB19*	SE	ST11	2
*qnrB19*	mSTM	ST19	3
*bla_TEM-1_*	SE	ST3233	1
*bla_TEM-1_*	STM	ST19	6
*bla_TEM-1_*	mSTM	ST34	1
*tetB*	mSTM	ST34	1
*aph(3”)-Ib*, *aph(6)-Id*, *sul2*, *tetA*	STM	ST19	3
*bla_TEM-1_*, *aph(3”)-Ib*, *aph(6)-Id*, *sul2*, *tetB*	mSTM	ST34	11
*bla_TEM-1_*, *aph(3”)-Ib*, *aph(6)-Id*, *aph(3′)-Ia*, *sul2*	mSTM	ST34	1
*bla_TEM-1_*, *aadA1*, *cmlA1*, *sul3*, *tetM*	STM	ST19	1
*bla_TEM-1_*, *aph(3”)-Ib*, *aph(6)-Id*, *sul2*, *tetB*, *qnrB19*	mSTM	ST34	1
*bla_TEM-1_*, *aph(3”)-Ib*, *aph(6)-Id*, *sul2*, *tetB*, *gyrA_S83F*	mSTM	ST34	1
*bla_TEM-1_*, *aph(3”)-Ib*, *aph(6)-Id*, *aph(3′)-Ia*, *sul2*, *tetB*	mSTM	ST34	1
*bla_TEM-1_*, *aph(3”)-Ib*, *aph(6)-Id*, *aadA1*, *sat2*, *sul2*, *tetB*, *dfrA1*	mSTM	ST34	1
*aac(3)-IVa*, *aadA1*, *aadA2*, *cmlA1*, *sul1*, *sul2*, *sul3*, *dfrA12*, *bleO*	mSTM	ST19	1
*bla_TEM-190_*, *aac(3′)-Ia*, *aph(3”)-Ib*, *aph(6)-Id*, *aadA5*, *mphA*, *floR*, *sul1*, *sul2*, *tetB*, *dfrA19*, *mcr-9.1*	mSTM	ST34	1
*bla_TEM-1_*, *aph(3”)-Ib*, *aph(6)-Id*, *aac(3)-IVa*, *aph(4)-Ia*, *ant(2”)-Ia*, *aadA1*, *aadA2*, *mefB*, *cmlA1*, *sul2*, *sul3*, *tetB*, *dfrA12*	mSTM	ST34	2

## Data Availability

The sequencing raw data presented in the study are openly available in the European Nucleotide Archive (ENA) under Project PRJEB40400 and PRJEB89723.

## Supplementary Materials

The following supporting information can be downloaded at: https://www.mdpi.com/article/10.3390/microorganisms14081716/s1. Figure S1: MBioSEQ Ridom Typer MST for 217 *Salmonella* Enteritidis Romanian genomes; Figure S2: MBioSEQ Ridom Typer MST for 54 *Salmonella* Typhimurium and monophasic *S.* Typhimurium Romanian genomes; Table S1: Metadata of the *Salmonella enterica* strains analyzed in this study and quality parameters of the obtained *draft* genomes; Table S2: Serovars, sequence types, complex types, and antimicrobial resistance profiles of the *Salmonella enterica* strains analyzed in this study; Table S3: Characteristics of the plasmids associated with the *Salmonella enterica* strains analyzed in this study.

## Abbreviations

The following abbreviations are used in this manuscript:

AMR	Antimicrobial Resistance
ECDC	European Centre for Disease Prevention and Control
WGS	Whole-Genome Sequencing
MLST	Multi-Locus Sequence Typing
cgMLST	Core Genome MLST
ST	Sequence Type
CT	Complex Type
EU	European Union
RC	Reference Center
ENA	European Nucleotide Archive
AD	Allelic Difference
SLV	Single-Locus Variant
MDR	Multi-Drug Resistant
ND	Not Determined
ASSuT	Ampicillin, Streptomycin, Sulfonamides, and Tetracyclines Resistant Profile
