# Supplementary material for: Genomic Insights into the Population Structure and Antimicrobial Resistance of the Most Prevalent Human-Associated Non-Typhoidal Salmonella Serotypes in Romania
Source: Microorganisms. 2026 Aug 5;14(8):1716. doi: 10.3390/microorganisms14081716 (PMC13515400; doi:10.3390/microorganisms14081716)
Supplement: Supplementary file 1 [file microorganisms-14-01716-s001.zip › Figure S1.pdf]

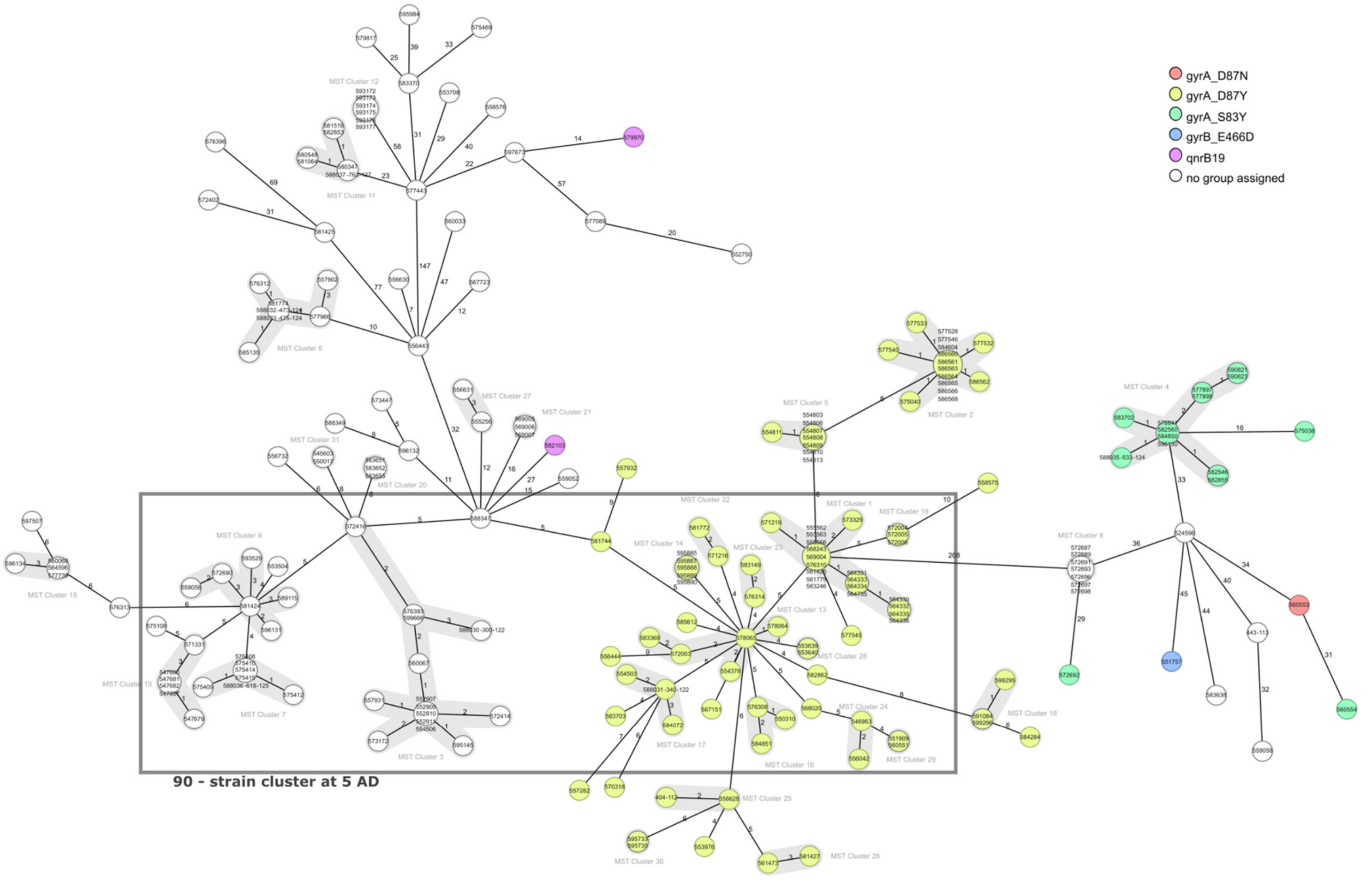

Figure S1. MBioSEQ Ridom Typer MST for 217 *Salmonella* Enteritidis Romanian genomes; distance based on *S*. enterica cgMLST; pairwise ignoring missing values; logarithmic scale; MST Cluster distance threshold 3 AD; nodes colored by quinolone resistance determinants
