# Supplementary material for: Genomic Insights into the Population Structure and Antimicrobial Resistance of the Most Prevalent Human-Associated Non-Typhoidal Salmonella Serotypes in Romania
Source: Microorganisms. 2026 Aug 5;14(8):1716. doi: 10.3390/microorganisms14081716 (PMC13515400; doi:10.3390/microorganisms14081716)
Supplement: Supplementary file 1 [file microorganisms-14-01716-s001.zip › Figure S2.pdf]

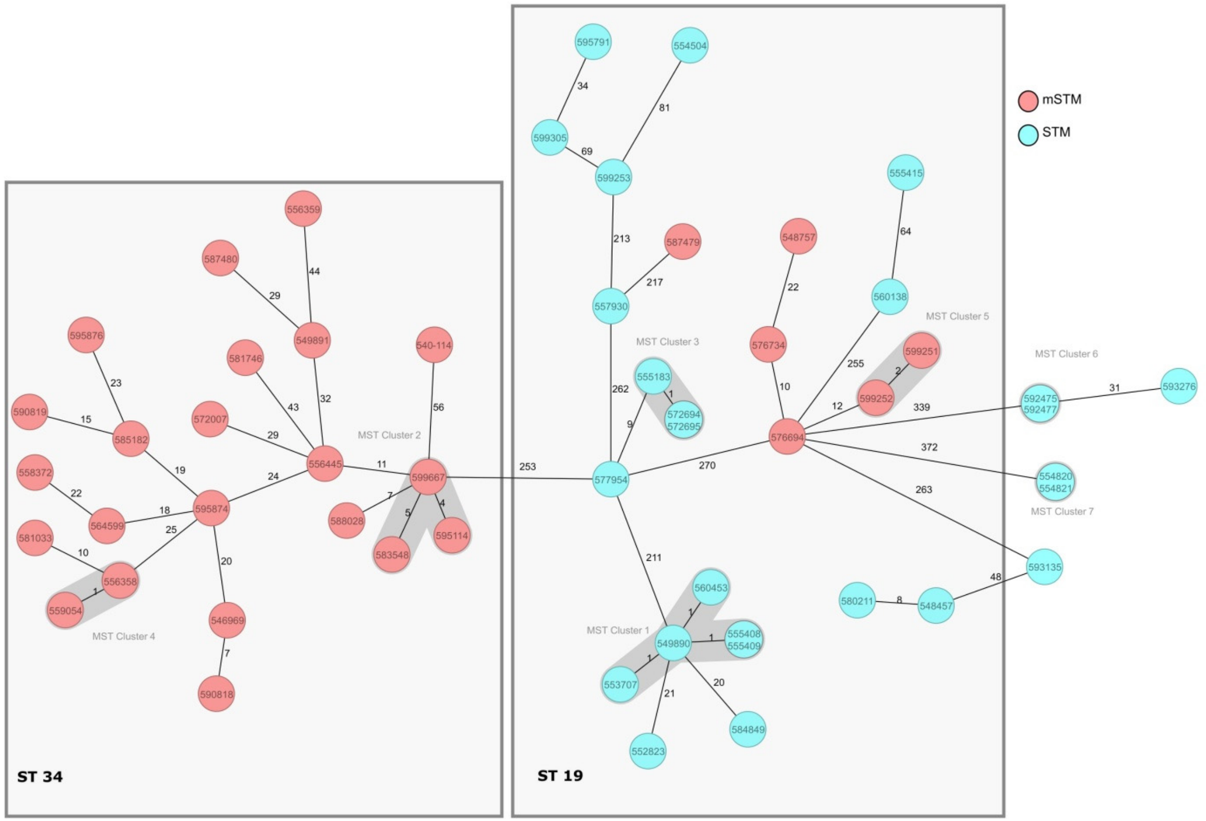

Figure S2. MBioSEQ Ridom Typer MST for 54 *Salmonella* Typhimurium and monophasic *S.* Typhimurium Romanian genomes; distance based on *S. enterica* cgMLST; pairwise ignoring missing values; logarithmic scale; MST Cluster distance threshold 5 AD; nodes colored by serovar.

STM = *S*. Typhimurium, mSTM = monophasic *S*. Typhimurium
